# CD44v6+ Hepatocellular Carcinoma Cells Maintain Stemness Properties through Met/cJun/Nanog Signaling

**DOI:** 10.1155/2022/5853707

**Published:** 2022-11-07

**Authors:** Wei Chen, Ronghua Wang, Yuchong Zhao, Yawen Li, Xiju Wang, Wang Peng, Shuya Bai, Mengli Zheng, Man Liu, Bin Cheng

**Affiliations:** ^1^Department of Gastroenterology and Hepatology, Tongji Hospital, Tongji Medical College, Huazhong University of Science and Technology, Wuhan, China 430030; ^2^Department of Surgery, University of Pittsburgh School of Medicine, Pittsburgh, PA, USA 15213; ^3^Department of Gastroenterology, Affiliated Hospital of Zunyi Medical University, Zunyi, Guizhou, China 563003; ^4^Department of Digestive Endoscopy, The Affiliated Hospital of Guizhou Medical University, Guiyi Street No. 28, Guiyang, Guizhou, China 550000; ^5^Department of Gastroenterology and Hepatology, Taikang Tongji Wuhan Hospital, Wuhan, China 430050

## Abstract

Cancer stem cells (CSCs) are characterized by their self-renewal and differentiation abilities. CD44v6 is a novel CSC marker that can activate various signaling pathways. Here, we hypothesized that the HGF/Met signaling pathway promotes stemness properties in CD44v6+ hepatocellular carcinoma (HCC) cells via overexpression of the transcription factor, cJun, thus representing a valuable target for HCC therapy. Magnetic activated cell sorting was used to separate the CD44v6+ from CD44v6- cells, and Met levels were regulated using lentiviral particles and the selective Met inhibitor, PHA665752. An orthotopic liver xenograft tumor model was used to assess the self-renewal ability of CD44v6+ cells in immunodeficient NOD/SCID mice. Luciferase reporter and chromatin immunoprecipitation assays were also conducted using cJun-overexpressing 293 T cells to identify the exact binding site of cJun in the Nanog promoter. Our data demonstrate that CD44v6 is an ideal surface marker of liver CSCs. CD44v6+ HCC cells express higher levels of Met and possess self-renewal and tumor growth abilities. Xenograft liver tumors were smaller in nude mice injected with shMet HCC cells. Immunohistochemical analysis of liver tissue specimens revealed that high Met levels in HCC cells were associated with poor patient prognosis. Further, a cJun binding site was identified 1700 bp upstream of the Nanog transcription start site and mutation of the cJun binding site reduced Nanog expression. In conclusion, the HGF/Met signaling pathway is important for maintenance of stemness in CD44v6+ HCC cells by enhancing expression of cJun, which binds 1700 bp upstream of the Nanog transcription start site.

## 1. Introduction

The incidence of hepatocellular carcinoma (HCC) has been rising worldwide. According to recent global cancer statistics, HCC ranks fifth as a cause of global cancer mortality, accounting for 8.2% of all cancer deaths [1, 2]. Although the survival rate for patients with HCC has increased due to improvements in surgical techniques and perioperative management, their prognosis remains poor. Liver tumor initiation and progression are driven by intracellular signaling pathways [3]; therefore, effective therapeutic strategies that disrupt cancer cell signaling pathways are required.

Tumor initiation, maintenance, and growth are driven by a small population of cancer cells termed cancer stem cells (CSCs), which can self-renew and differentiate into multiple cell types [4]. CSCs possess various phenotypes associated with therapeutic resistance and often cause disease recurrence [4], and cancer-associated mortality is primarily caused by cancer recurrence and metastasis. CD44, as a transmembrane receptor for hyaluronic acid, has been used as a cell surface marker of mammalian CSCs [5]. Posttranslation modifications and alternative splicing can affect the binding affinity of CD44 for its ligands and regulate CSC activities. Further, there is accumulating evidence that CD44v isoforms have critical roles in regulating stemness properties, including self-renewal, tumor initiation, metastasis promotion, and chemotherapy resistance in prostate [6], colon [7, 8], intestinal [9], and gastric [10] cancer cells [11]. CD44v6 is a marker of constitutive and reprogrammed CSCs that drive colon cancer metastasis, and low levels of CD44v6 predict better prognosis [12]. The specific mechanism by which the CD44v6 regulates stemness properties of cancer cells is unclear; however, CSCs are associated with cancer malignancy. Therefore, therapies that target CD44v6 may destroy CSC populations, representing a promising potential strategy for treatment of life-threatening cancers.

Met is a receptor tyrosine kinase (RTK) which binds to hepatocyte growth factor (HGF) to activate a wide range of different cellular signaling pathways, including those involved in proliferation, motility, migration, and invasion [13]. HGF acts as a pleiotropic factor and cytokine, promoting cell proliferation, survival, motility, scattering, differentiation, and morphogenesis [14–16]. HGF binding to Met results in receptor homodimerization and phosphorylation of two tyrosine residues located within the catalytic loop of the tyrosine kinase domain [17]. The tyrosine residues recruit signaling effectors, including mitogen-activated protein kinase (MAPK) cascades, the phosphoinositide 3-kinase–Akt (PI3K–Akt) axis, and signal transducer and activator of transcription proteins (STATs) [18, 19]. Together, these observations indicate that Met activation could be among the molecular mechanisms responsible for maintenance of cancer cell stemness.

CSCs can express several unique transcription factors to preserve their stemness, such as Nanog, Sox2, Oct4, and Klf4. As a crucial transcription factor regulating CSC growth that also functions in embryogenesis and tumorigenesis, Nanog can induce self-renewal, metastasis, tumorigenesis, and drug-resistance in CSCs [20]. High Nanog expression is strongly associated with advanced stage and low overall patient survival rate in various cancers [21], in which Nanog can regulate the stemness properties of CSCs through various signaling pathways. For example, the Nanog/Tcl1a/Akt signaling axis [22, 23], epithelial-mesenchymal transition- (EMT-) associated signaling [24, 25], and the Nanog/MAPK/Erk1 signaling pathway [26] are all involved in regulation of CSC activities. Thus, Nanog is a crucial target to increase the efficacy of HCC CSC eradication during cancer treatment.

In this study, we demonstrate that CD44v6 can serve as an ideal surface marker of liver CSCs. Further, we present a comprehensive analysis of the mechanism underlying maintenance of the stemness properties of CD44v6+ HCC cells via the HGF/MET signaling pathway and provide the first evidence of for a specific cJun binding site. These findings are crucial to understanding the role of CSCs in carcinogenesis.

## 2. Methods

### 2.1. Patient Liver Tissue Specimens and Demographic Information

Paraffin-embedded sections of HCC and adjacent liver specimens were obtained from 53 patients with HCC that underwent curative resection between 2013 and 2016 at the Tongji Hospital, Huazhong University of Science and Technology (HUST, Wuhan, China). Clinical data associated with these specimens were recorded without patient identification. All human experiments were approved by the ethics committee of Tongji Hospital. Informed consent was obtained from all subjects. Patients were enrolled as described previously [27]. Tumor differentiation was defined according to the Edmondson grading system [28].

### 2.2. Cell Lines and Cell Culture

The human HCC cell lines, PLC/PRF/5 and SNU398, were obtained from the American Type Culture Collection (Manassas, VA, USA) and the Cell Bank of the Chinese Academy of Sciences (Shanghai, China), respectively. Met knockout and overexpression cell lines were generated in our laboratory. All cell lines were cultured in Dulbecco's Modified Eagle's Medium (DMEM; GIBCO, Grand Island, NY, USA) supplemented with 10% fetal bovine serum (FBS; GIBCO) at 37°C and 5% CO_2_. Cells were confirmed to be free of mycoplasma using the Bimake mycoplasma detection kit.

### 2.3. Mice and Tumor Models

Four-week-old male NOD/SCID and BALB/c nude mice were obtained from Beijing Huafukang Biotechnology Company and maintained under pathogen-free conditions. Animal experiments were performed according to the NIH Guide for the Care and Use of Laboratory Animals, with the approval of Tongji Hospital Institutional Review Board. All mice used in our experiments were sex and age matched (range, 6–10 weeks). To generate orthotopic liver xenograft tumor models, 1 × 10^5^ luciferase-labeled SNU398 cells were injected into the left lobes of the liver; animals were sacrificed 4–5 weeks after implantation. Bioluminescence was measured 5 min after tail intravenous injection administration of 100 *μ*l potassium D-luciferin salt (30 mg/ml) dissolved in PBS (per animal). For the subcutaneous xenograft tumor model, 1 × 10^5^ CD44v6+/− cells were injected subcutaneously in the shaved right flanks of mice. Tumors were surgically removed when they reached 1000 mm^3^ (time range, 25–28 days). Tumor growth was followed by measurement with calipers; tumor volume = *xy*^2^/2, where *x* is the longest and *y* the shortest of two perpendicular diameters.

### 2.4. Cell Migration and Invasion Assays

Cell invasion and migration abilities were assessed using 8 *μ*m pore, 6.5 mm polycarbonate transwell filters (Millipore, Billerica, MA, USA), as described previously [29]. For the migration assay, 5 × 10^4^ cells were seeded on the upper chamber with serum-free DMEM. DMEM supplemented with 10% FBS was added to the lower chamber. After incubation for 36–48 h, cells were fixed with paraformaldehyde and stained with 4 g/l crystal violet solution for 0.5 h. Cells were counted using a microscope. For the cell invasion assay, chambers were uniformly coated with a Matrigel layer; subsequent procedures were the same as those for the migration assay. Migration and invasion experiments were performed in triplicate.

### 2.5. Spheroid-Forming Assay

Spheroid-forming assays were conducted as previously described [29], with minor modification. Cells lines were plated in 24-well, ultra-low attachment plates (Corning, NY, USA) at a density of 3000 viable cells per well. Cells were grown in spheroid medium consisting of DMEM/F12 medium (cat. #12400-024; GIBCO) supplemented with 20 ng/ml human recombinant epidermal growth factor (cat. #PHG0311; GIBCO), 10 ng/ml human recombinant basic fibroblast growth factor (cat. #PHG0266; GIBCO), 100 IU/ml penicillin, 100 *μ*g/ml streptomycin, 2% B27 supplement (cat. #17504-044; GIBCO), 1% N-2 supplement (cat. #17502-048; GIBCO), and 1% methyl cellulose (cat. #M0262; Sigma-Aldrich, St. Louis, MO, USA), to prevent cell aggregation. Spheres containing over 100 cells were counted. The experiment was terminated after 10–12 days, and the spheroids quantified.

### 2.6. Cell Counting Kit-8 (CCK8) Cytotoxicity Assay

The sensitivity of cells to sorafenib was measured by CCK8 assay. Cells were seeded in 96-well plates at a density of 1000 cells per well, then treated with 1 *μ*m sorafenib (Sigma-Aldrich) after the cells attached to the culture vessels. Cells were then incubated for 24 h, the medium replaced with fresh culture medium, CCK8 (Promoter, China) added to each well according to the manufacturer's protocol, and incubated at 37°C for 2 h. Absorbance was measured at 450 nm using a microplate reader (Thermo Scientific).

### 2.7. RNA Isolation and Reverse Transcription Quantitative PCR (RT-qPCR)

Total RNA was extracted from spheroids or cells using TRIzol®, according to standard protocols (https://assets.thermofisher.com/TFS-Assets/LSG/manuals/trizol_reagent.pdf). Real-time quantitative PCR was performed using SYBR Premix (DRR041A, TaKaRa, Japan), as described previously [30]. A standard curve for each gene was generated from serially diluted standards, and values for unknown samples were extrapolated. *β*-Actin was used as an internal control to normalize samples. All standards and samples were assayed in triplicate. The assay details and primer sequences are presented as Supplementary Material (Table S1).

### 2.8. Western Blot Analysis

Western blot analysis was performed as described previously [29]. Primary antibodies for cJun (cat. #9165), Nanog (cat. #4903), Met (cat. #8198), Erk (cat. #4695), and p-Erk (cat. #4370) were purchased from Cell Signaling Technology (CST), Inc. (Danvers, MA, USA). Antibody against CD44v6 (cat. #ab30436, Abcam) was purchased from Abcam, and those recognizing Snail1 (cat. #13099-1-AP), Slug (cat. #12129-1-AP), and Twist (cat. #25465-1-AP) were purchased from Proteintech Group Inc. Monoclonal mouse anti-*β*-actin (cat. #ab8226; Abcam) was used as an internal control.

### 2.9. Immunohistochemistry (IHC)

IHC staining with antibodies against CD44v6 (Abcam, ab78960), Nanog (CST, cat. #4903), Met (CST cat. #8198), and Ki67 (CST, cat. #9449) was performed to detect protein expression levels, which were calculated according to staining intensity and positive staining. Then, patients were divided into two groups with low and high expression for survival analysis. Assessment of IHC staining scores was performed independently by two pathologists who were blinded to the clinical data.

### 2.10. Cell Sorting

CD44+, CD44-, CD44v6+, and CD44v6- cells were obtained by magnetic activated cell sorting (MACS). MACS sorting of the CD44 and CD44v6 epitopes was conducted using a PE-conjugated anti-human antibody and EasySep™ Human PE Positive Selection Kit II (STEMCELL Technologies, Vancouver, Canada), according to the standard protocol (https://cdn.stemcell.com/media/files/pis/DX22359-PIS_1_0_0.pdf). CD44v6+ LCSCs were cultured as described previously [30].

### 2.11. Flow Cytometry

Cultured cells (5 × 10^5^) were centrifuged, and the cell pellets suspended in FACS buffer (PBS containing 0.5% FBS), then labeled with PE-conjugated human CD44v6 antibody (R&D Systems, #FAB3660P) (4°C, 30 min). Cells were washed twice, resuspended in FACS buffer and analyzed using a FACSCalibur machine with CellQuest software (BD Biosciences).

### 2.12. Luciferase Reporter Assay

To construct luciferase vectors containing Nanog promoter sequences, three different Nanog promoter sequences were amplified and inserted into a luciferase reporter plasmid before the Kozak and luciferase sequences (P1–P3: −2200–0, −1700–0, and −700–0 bp upstream of the Nanog coding sequence, respectively) using VectorBuilder services (VectorBuilder Inc., Chicago, IL, USA). The cJun-expressing and luciferase reporter plasmids were cotransfected into cells; empty vectors were used as controls. Luciferase signals were detected using a luciferase reporter assay kit (Promega, Madison, Wisconsin, USA) and measured using a BMG FLUOstar OPTIMA Microplate Reader (BMG LABTECH, Cary, North Carolina, USA).

### 2.13. Chromatin Immunoprecipitation (ChIP)

ChIP assays were performed using a SimpleChIP Plus Enzymatic Chromatin IP kit (Cell Signaling Technology, #38191) with minor modifications. Spheroid cells (10^7^) were crosslinked with 1% formaldehyde for 5 min. Glycine was then added to a final concentration of 0.125 M for 5 min. Chromatin was disrupted by the addition of 0.5 *μ*l Micrococcal Nuclease per IP prep (20 min, 37°C) with frequent mixing. Three pairs of primers for potential cJun binding sites were designed; the assay details and primer sequences are presented in Supplementary Methods (Table S2).

### 2.14. Promoter Prediction Analysis

Three promoter prediction websites were used to locate putative cJun binding sites in the Nanog promoter region, including Promo (http://alggen.lsi.upc.es/cgibin/promo_v3), TFbinding (http://tfbind.hgc.jp), and JASPAR (http://jaspar.genereg.net); JASPAR provides specific predicted binding sequences.

### 2.15. Gene Set Enrichment Analysis (GSEA)

Gene expression profiles and clinical data from patients with HCC were obtained from The Cancer Genome Atlas (TCGA) data portal (https://www.cancer.gov/tcga/) in December 2020. HCC samples were divided into low and high Met expression groups, using the median value as the cutoff. GSEA v4.0.1 was applied to perform GO and KEGG analyses to investigate the biological functions and pathways in HCC pathogenesis involving Met.

### 2.16. Statistical Methods

Data are presented as mean ± SD. Statistical significance was considered as *p* < 0.05. The significance of differences was assessed by one-way analysis of variance (ANOVA) or Student's *t*-test.

## 3. Results

### 3.1. CD44v6+ HCC Cells Possess Stemness Properties

**Figure 1:**
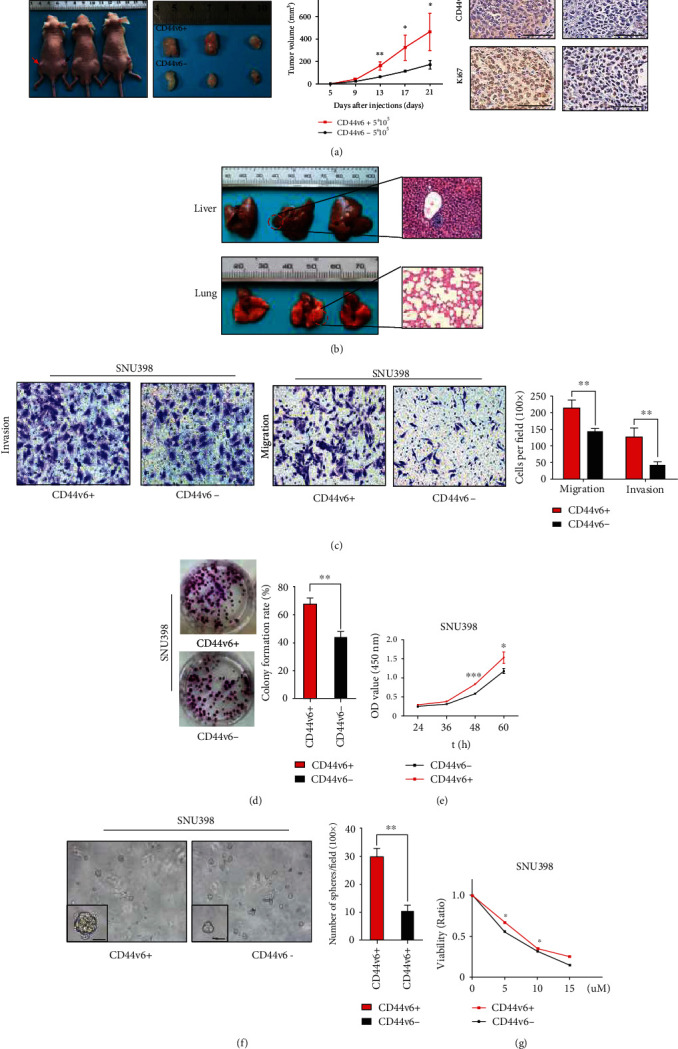
CD44v6+ HCC cells possess stemness properties. (a) CD44v6 negative/positive SNU398 cells were subcutaneously injected into the BALB/c mice. Red arrow means CD44v6 positive group, black arrow means negative group. The tumor volume from each group were tested. For statistical analysis, ^∗^*p* < 0.05, ^∗∗^*p* < 0.01, *t*-test. The representative images of IHC staining of CD44v6 and Ki67 in CD44v6 positive and negative group tumor tissues. Bars: 200 *μ*m. (b) The liver and the lung of the SNU398 cell subcutaneously treated mice were dissected. Red circle indicates the site of metastatic tumor. Hematoxylin-eosin (HE) staining showed the metastatic tumor in the liver. (c) Representative images of transwell migration and invasion in indicated cells. Scale bar, 200 *μ*m. ^∗∗^*p* < 0.01, *t*-test. (d) The colony formation assay showed that the colony formation ability of CD44v6+ cells was stronger than CD44v6- cells. ^∗∗^*p* < 0.01, *t*-test. (e) CCK8 assay showed the cell proliferation in CD44v6+ cells was stronger than CD44v6- cells. ^∗^*p* < 0.05, ^∗∗∗^*p* < 0.001, *t*-test. (f) The representative images of spheres and histogram analysis in indicated cells. Scale bar, 200 *μ*m. ^∗∗^*p* < 0.01, *t*-test. (g) Cell viability assay was performed using CCK8 assay in SNU398 cells, which were treated with indicated concentrations of sorafenib for 24 hours. And the number of viable cells represented the susceptibility of the CD44v6+ cells to antisorafenib. ^∗^*p* < 0.05, *t*-test.

Our previous studies have demonstrated that patients with HCC with high CD44v6 expression have shorter overall and disease-free survival than those of patients with low CD44v6 expression [30]. To investigate the tumorgenicity of CD44v6+ CSCs in vivo, CD44v6+ and CD44v6− cells isolated from the HCC cell line, SNU398 (Figure S1A), were injected subcutaneously into 4-week-old male BALB/c nude mice. As expected, the mean volume of tumor nodules in the CD44v6+ group was significantly higher than that in the CD44v6− group (Figure 1(a); day 21 tumor volume, 462.9 ± 95.62 mm^3^ vs. 169.2 ± 22.36 mm^3^, ^∗^*p* < 0.05, *t*-test). In addition, we conducted Ki67 IHC staining of subcutaneous xenograft tumors from mice to explore the proliferation ability of CD44v6+ and CD44v6- HCC cells and found that proliferation ability was lower in the CD44v6− group. Interestingly, we also found a CD44v6+ tumor metastasis in the liver of a BALB/c nude mouse following subcutaneous tumor cell injection (metastasis tumor volume, 54 mm^3^), while there were no metastases in the CD44v6− group (Figure 1(b)).

As reported in colorectal cancer [8], CD44v6+ HCC cells exhibited significantly higher migration and invasion ability than CD44v6− cells (Figure 1(c); ^∗∗^*p* < 0.01, *t*-test). We then compared the proliferative ability of cancer cells using a colony formation assay. The results indicated that CD44v6+ cells possessed enhanced self-renewal ability relative to CD44v6− cells (Figure 1(d); ^∗∗^*p* < 0.01, *t*-test). The CCK8 proliferation assay also produced consistent results (Figure 1(e); ^∗^*p* < 0.05, ^∗∗∗^*p* < 0.001, *t*-test). Further, in sphere formation assays, there were significantly more sphere clones in CD44v6+ than CD44v6- SNU398 cell line subsets, and the number of spheres with diameter ≥ 100 *μ*m was 28.67 ± 2.028/HP and 5.667 ± 0.6667/HP in CD44v6+ and CD44v6- cells, respectively (Figure 1(f); ^∗∗∗^*p* < 0.005, *t*-test). In addition, CD44v6+ cells were more resistant to sorafenib (a MAPK/ERK multityrosine kinase inhibitor) than their CD44v6− counterparts (Figure 1(g); ^∗^*p* < 0.05, *t*-test). Collectively, these data indicate that CD44v6 is a promising surface marker for liver CSCs.

### 3.2. The HGF/MET Signaling Pathway Contributes to Maintenance of Stemness in CD44v6+ HCC Cells

Previous studies have reported that HGF/MET pathway activation is required for CD44v6 activity [31, 32]. Mature HGF/MET and CD44v6 proteins form a complex, and Met activation, as well as complex formation, is prevented by antibodies recognizing the v6 epitope or other epitopes in the membrane-proximal stem structure of CD44 [33]. In this part of study, we analyzed potential associations between the CD44v6 and Met molecules.

**Figure 2:**
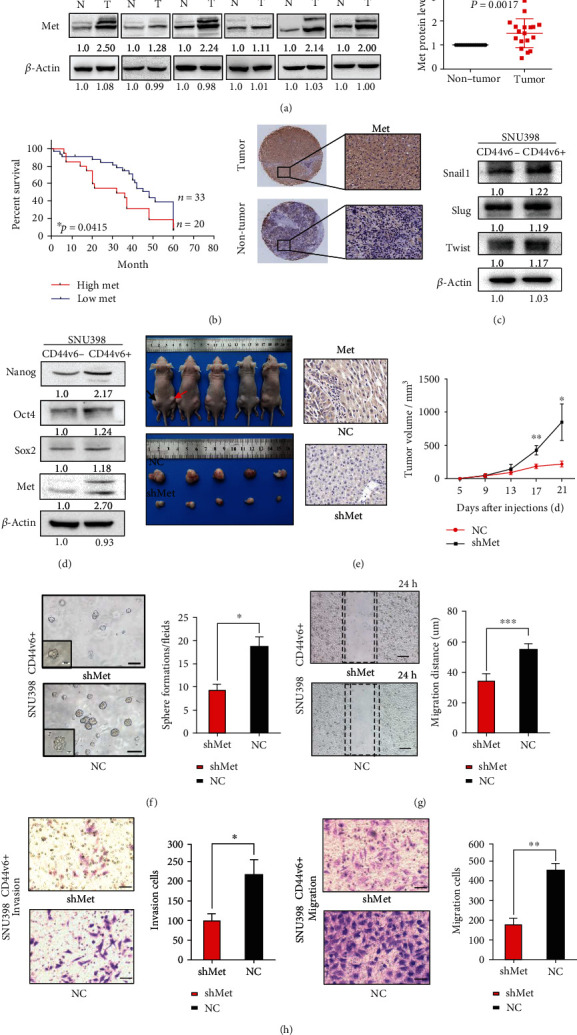
The HGF/MET signaling pathway contributes to maintenance of stemness in CD44v6+ HCC Cells. (a) Western blot analysis of the Met protein levels in 18 paired HCC tissues and adjacent nontumor tissues selected randomly. *β*-Actin was used as a normalized control. (b) Kaplan–Meier survival analysis of overall survival were compared according to the expression levels of the Met in HCC tissues. Patients with Met expression median survival = 32 months vs. 48 months, log-rank test, *n* = 53, ^∗^*p* = 0.0415. The immunohistochemical staining shows the expressions of Met in surgical specimen from patients with HCC. The expression of Met in tumor tissue was significantly higher than in adjacent nontumor tissue. Scale bar, 20 *μ*m. (c) The expression of EMT-related genes, including Snail1, Slug, and Twist in CD44v6+ and CD44v6- cells. *β*-Actin was used as a normalized control. (d) The expression of Met and the stemness relative genes, including Nanog, Sox2, and Oct4 in CD44v6+ and CD44v6- cells. *β*-Actin was used as a normalized control. (e) shMet CD44v6+ cells and scrambled cells were subcutaneously injected into the BALB/c mice (*n* = 5 in each group). Black arrow means NC group, red arrow means Met shRNA group. The tumor volume from each group was tested. For statistical analysis, ^∗^*p* < 0.05, ^∗∗^*p* < 0.01, *t*-test. The representative images of IHC staining of Met in NC and shMet group tumor tissues. Bars: 200 *μ*m. (f) The representative images of spheres and histogram analysis in indicated cells. Scale bar, 200 *μ*m. ^∗^*p* < 0.05, *t*-test. (g) The representative images of the wound-healing experiment and histogram analysis in indicated cells. Scale bar, 100 *μ*m. ^∗∗∗^*p* < 0.001, *t*-test. (h) Representative images of transwell migration and invasion in indicated cells. Scale bar, 200 *μ*m. ^∗^*p* < 0.05, ^∗∗^*p* < 0.01, *t*-test.

First, we examined Met protein expression by western blot analysis of 18 paired tumor and para-tumor samples from patients with HCC. Met expression was significantly higher in HCC than in paratumor tissues (Figure 2(a)). Next, to evaluate the prognostic value of Met in human HCC tissues, we examined their expression in a tissue microarray comprising 53 paired tissue samples from patients with HCC by IHC. Patients with HCC with higher Met expression had shorter overall survival than those with low Met expression (Figure 2(b); median survival = 40 months vs. 55 months, ^∗^*p* < 0.05, log-rank test). Representative images of IHC staining of Met, CD44v6, and Nanog are presented in Figure 2(b) and Figure S1B. In summary, these results indicate that higher Met expression is associated with poor prognosis in patients with HCC.

Second, to explore the role of HGF/MET signaling in maintenance of CD44v6+ HCC cell stemness properties, we performed cell sorting experiments using the SUN398 cell line and examined the expression of EMT-related genes in CD44v6+ and v6− cells. Expression levels of Snail1, Slug, and Twist were significantly higher in CD44v6+ cells (Figure 2(c), Figure S1C). Next, we evaluated the expression of Met and the stemness-related factors, Nanog, Sox2, and Oct4, in CD44v6+ and v6− cells (Figure 2(d)). Nanog protein was significantly overexpressed in CD44v6+ cells and consistent results were generated by RT-qPCR experiments (Figure S1D). Our in vivo data showed that the volume of tumors derived from Met shRNA CD44v6+ cells in BALB/c nude mice was smaller than that of tumors derived from cells treated with scrambled control shRNA in a subcutaneous xenograft tumor model (Figure 2(e); day 21 tumor volume, 848.6 ± 157.0 mm^3^ vs. 221.0 ± 23.03 mm^3^, ^∗^*p* < 0.05, ^∗∗^*p* < 0.01, *t*-test). Representative images of samples IHC stained for Met are shown in Figure 2(d). Further, we found that knocking down the HGF/MET signaling pathway also decreased Nanog expression, as detected by IHC staining (Figure S1E).

Third, we used PHA665752, a selective Met inhibitor, to suppress Met activation in CD44v6+ and CD44v6− HCC cells. Western blot assays showed that Met and AKT levels were significantly repressed (Figure S1F). Further, expression of the stemness-related genes, Nanog, Oct4, and Sox2, were significantly reduced in CD44v6+ HCC cells (Figure S1G), as were those of EMT-related genes (Figure S1H). Moreover, our results show that knocking down Met using lentivirus interfered with sphere colony formation capacity (Figure 2(f); 18.67 ± 2.028/HP vs. 9.333 ± 1.202, ^∗^*p* < 0.05, *t*-test), wound healing migration ability (Figure 2(g); 34.50 ± 1.607 *μ*m vs. 55.00 ± 1.528 *μ*m, ^∗^*p* < 0.05, *t*-test), and transwell invasion and migration capacity (Figure 2(h); invasion: 225.7 ± 24.50/HP vs. 108.0 ± 10.82/HP, migration: 452.3 ± 41.34/HP vs. 176.7 ± 35.31/HP, ^∗^*p* < 0.05, *t*-test). Together, our data demonstrate that CD44v6/MET signaling contributes to maintenance of stemness of liver CSCs.

### 3.3. The HGF/MET Signaling Pathway Activates Nanog Expression in CD44v6+ HCC Cells

**Figure 3:**
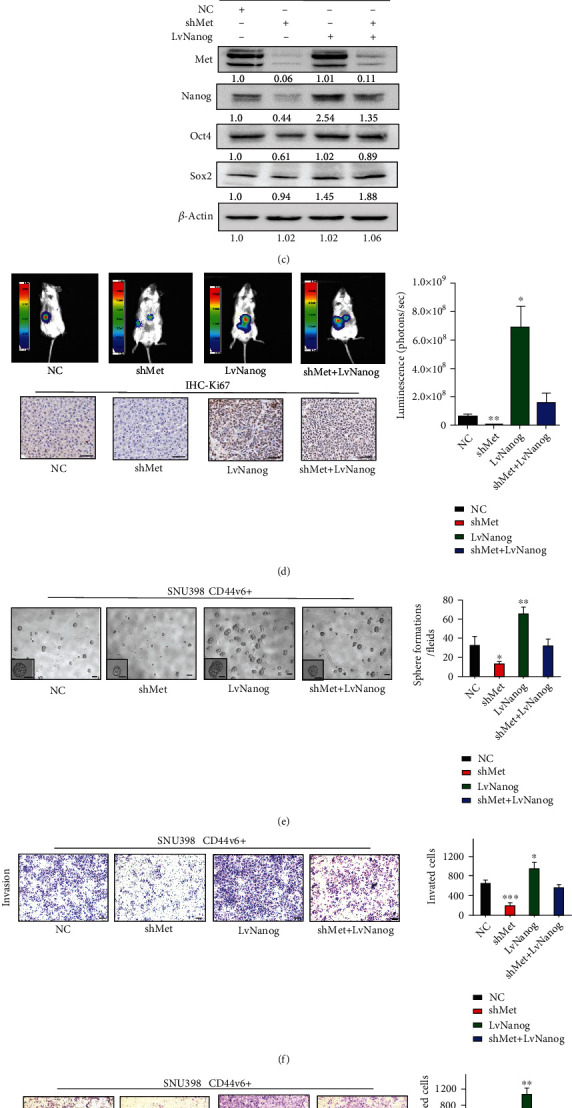
The HGF/MET signaling pathway activates Nanog expression in CD44v6+ HCC cells. (a) Silencing Met decreased the expression of stemness relative genes, including Oct4, Sox2, and Nanog in CD44v6+ SNU-398 cells. *β*-Actin was used as a normalized control. (b) The expression of EMT-related genes, including Snail1, Slug, and Twist in CD44v6+ SNU-398 cells. *β*-Actin was used as a normalized control. (c) Western blot showed that silencing Met decreased the expression of Nanog in CD44v6+ cells. But LvNanog did not affect the Met expression. *β*-Actin was used as a normalized control. (d) 1 × 10^5^ of cells were injected into the left lobes of the liver. Bioluminescence signals from Met shRNA groups were weaker than that from corresponding control groups. But overexpressed Nanog could rescue the bioluminescence signals. The central luminescence intensity: 8.8e + 7 vs. 1.6e + 6; ^∗^*p* < 0.05, ^∗∗^*p* < 0.01; *t*-test. The representative images of Ki67 immunohistochemistry. Scale bar, 50 *μ*m. (e) The representative images of spheres and histogram analysis in indicated cells. Scale bar, 200 *μ*m. (f) and (g) transwell migration and invasion assays showed the knockdown of Met decreased the migration and invasion of CD44v6+ cells, while overexpressed Nanog could rescue the migration and invasion ability. Scale bar, 200 *μ*m. ^∗^*p* < 0.05, ^∗∗^*p* < 0.01, ^∗∗∗^*p* < 0.001; *t*-test.

To further explore the function of the HGF/MET signaling pathway, we transfected Met shRNA into SNU398 CD44v6+ cells to knock down Met expression. We found that Met knockdown decreased the expression of stemness-related proteins, particularly Nanog (Figure 3(a)) and ERK/p-ERK (Figure S2A); RT-qPCR results were consistent (Figure S2B). When Met expression was disturbed, EMT-related genes were simultaneously downregulated (Figure 3(b), Figure S2C). When we transfected LvNanog lentivirus and shMet lentivirus into HCC cells subsequently, we found that overexpression of Nanog in HCC cells with shMet knockdown did not rescue Met expression (Figure 3(c)). These data suggest that Nanog is downstream of the HGF/MET pathway.

To confirm the synergistic effect of Met and Nanog in regulating the stemness properties (including self-renewal and tumorigenic capacity) of CD44v6+ CSCs, we generated a mouse orthotopic liver xenograft tumor model in NOD/SCID mice. Luminescence intensity from the Met shRNA cells group was weaker than that derived from cells treated with scrambled control shRNA (Figure 3(d); central luminescence intensity: 8.8e + 7 vs. 1.6e + 6; ^∗∗^*p* < 0.01, ^∗^*p* < 0.05; *t*-test). Further, luminescence intensity was rescued following LvNanog transduction of Met shRNA cells (Figure 3(d); central luminescence intensity: 1.6e + 6 vs. 2.9e + 8; *p* = 0.083; *t*-test). Ki67 IHC analysis showed that LvNanog orthotopic liver xenograft tumors had stronger proliferation capacity. Next, we performed a spheroid-forming assay (Figure 3(e); ^∗^*p* < 0.05; *t*-test) and transwell migration and invasion experiments (Figure 3(f) and 3(g); ^∗^*p* < 0.05, ^∗∗^*p* < 0.01, ^∗∗∗^*p* < 0.001; *t*-test). The results showed that knocking down HGF/MET signaling reduced cell migration, invasion, and proliferation capacity. More interestingly, when we subsequently overexpressed Nanog, cell migration, invasion capacity, proliferation, and colony formation capacity were rescued. The above findings demonstrate that Nanog is a key downstream effector protein of the HGF/MET pathway in CD44v6+ cells.

### 3.4. The Transcription Factor, cJun, Mediates HGF/MET Signaling and Regulates Nanog Expression

**Figure 4:**
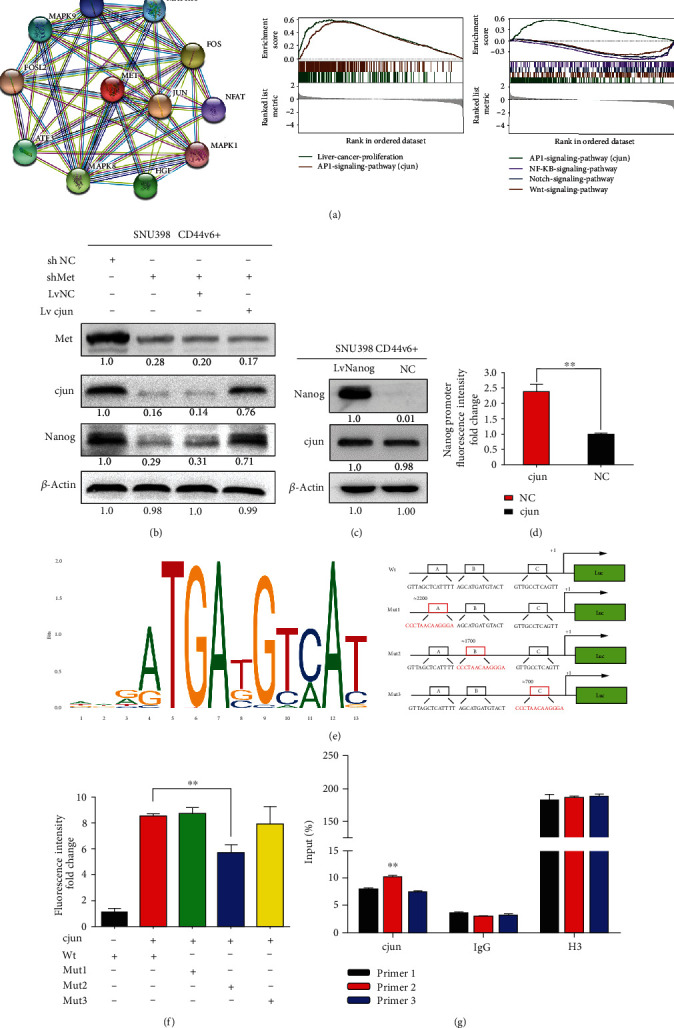
The transcription factor, cJun, mediates HGF/MET signaling and regulates Nanog expression. (a) STRING (https://string-db.org/cgi/input.pl) analysis showing functional association networks of cJun and Met-correlated genes. Gene Set Enrichment Analysis (GSEA) was used to analyze the expression of Met associated with the transcription factor cJun. (b) Immunoblotting analysis results showed that the expression of Nanog recovered after knockout of Met and transferred to cJun in the meantime. *β*-Actin was used as a normalized control. (c) Immunoblotting analysis showed the expression of cJun was barely changed when we overexpressed the Nanog. *β*-Actin was used as a normalized control. (d) Luciferase reporter assay analysis of Nanog promoter luciferase reporters in SNU398 cells before luciferase detection. It showed that there is a cJun binding site in the 0–2000 bp upstream regions of Nanog promoter. (e) The prediction of the binding sites of cJun to DNA in Jaspar Database (http://jaspar.genereg.net). And the schematic diagram shows three luciferase reporters cover different DNA sequences of Nanog promoter region. (f) Luciferase reporter assay analysis of three Nanog promoter luciferase reporters in SNU398 cells transfected with cJun or pcDNA. ^∗∗^*p* < 0.01, *t*-test. (g) ChIP assay showed that primer2 is the exact binding site of cJun. ^∗∗^*p* < 0.01.

cJun and TCF4/*β*-catenin are reported to interact on the Nanog promoter in vivo [34], and our bioinformatics analyses indicated that the cJun transcription factor is related to the HGF/MET signaling pathway (Figure 4(a)). Next, we used GSEA v4.0.1 to perform Gene Ontology (GO) and Kyoto Encyclopedia of Genes and Genomes (KEGG) analyses of HCC sample data from TCGA database. Our analysis demonstrated that the HGF/MET pathway was associated with liver cancer cell proliferation and that Met expression was involved in regulation of the compound transcription factor, AP1 (comprising cJun, cFos, and ATF) (Figure 4(a)). To further explore this mechanism, we used western blotting to detect cJun expression in shMet CD44v6+ and control cells (Figure 4(b)) and found that it was decreased in the shMet group, as was Nanog expression. We then examined cJun expression in LvNanog CD44v6+ and control cells. As expected, cJun expression did not differ significantly between these groups (Figure 4(c)). In conclusion, we found that cJun was downstream of HGF/MET signaling and regulates Nanog expression.

Therefore, we hypothesized that cJun binds to the Nanog promoter region and increases its expression. To investigate whether Nanog is a transcription target of cJun in CD44v6+ CSCs, we conducted luciferase reporter assays using vectors containing regions from 0–2000 bp of the Nanog promoter. The luciferase reporter assay data revealed that cJun overexpression in 293 T cells significantly increased Nanog luciferase reporter activity, compared with empty vector controls (Figure 4(d); ^∗∗^*p* < 0.01, *t*-test). To determine the exact binding site, we analyzed regions upstream of the Nanog transcription start site (TSS). As shown in Figure 4(e), we identified three predicted cJun binding sites (A, B, and C) on the Nanog promoter and determined the predicted binding sequence. Next, we designed three pGL3-based luciferase reporter constructs (P1–P3) containing Nanog promoter regions with mutations of the potential binding sites (cluster Mut 1–3), based on the three predicted cJun binding sites on the Nanog promoter. Our results show that 293 T cells transfected with Mut2 plasmid exhibited significantly lower luciferase activity than those transfected with Mut1 and Mut3 plasmids (Figure 4(f); ^∗∗^*p* < 0.01; *t*-test). These results strongly suggest the presence of a functional cJun binding site on the Nanog promoter localized in the cluster 2 region (approximately 1700 bp upstream of the Nanog TSS, with sequence AGCATGATGTACT) that plays an active role in cells.

To probe the direct binding of cJun to the site in the Nanog promoter region in the natural chromatin context of HCC, we next conducted a ChIP assay; the nucleotide sequences of the primers used are provided in the methods section. DNA pull-down using beads containing cJun antibody was used as a template for an RT-PCR using primers specific for the Nanog promoter site and demonstrated that pull-down of the DNA region amplified using Primer 2 was significantly higher than those of the regions amplified using Primers 1 or 3 (Figure 4(g); ^∗∗^*p* < 0.01; *t*-test). Our data indicate that the pulled down DNA amplified with Primer 1 after transformation with cJun expression vectors was significantly higher. Overall, our findings demonstrate that the cJun transcription factor can activate Nanog by binding directly to a site 1700 bp upstream of the Nanog TSS, with the sequence, AGCATGATGTACT.

## 4. Discussion

In this study, we demonstrate that CD44v6+ CSCs not only facilitate cancer stemness properties, such as self-renewal, tumor initiation, promotion of metastasis, and chemotherapy resistance, but also express high levels of the HGF/MET pathway. HGF/MET signal activation induced Met protein expression and promoted activation of downstream signaling pathways, such as the MAPK pathway. Inhibiting the activation of Met in CD44v6+ HCC cells in vivo and in vitro using specific inhibitors and lentiviruses led to a significant decrease in expression of the downstream transcription factor, cJun, and the stemness-associated gene, Nanog. Cell migration and invasion capacities were also impaired by Met knockdown in NOD/SCID mouse subcutaneous tumor and orthotopic liver xenograft tumor models. Moreover, we designed plasmids containing the wild-type Nanog promoter and three mutated promoter sequences to explore the exact cJun binding site. Further, luciferase reporter and the ChIP assays demonstrated that cJun could bind to a specific site 1700 bp upstream of the Nanog TSS and that Nanog expression was downregulated when this site was mutated.

Various pieces of evidence support essential roles for CSCs in tumorigenesis and drug-resistance. Epithelial cell adhesion molecules in human HCC cell lines are targets of chemoresistance [35, 36], while CD133 [37, 38], CD13 [39, 40], CD44 [41], OV-6 [42], and ALDH [43] are widely recognized as liver CSC surface markers. Among these surface markers, detection of the expression levels of circulating CD44 splice variant (CD44v) mRNA molecules in blood samples from patients with HCC can be used as an adjuvant method of predicting and monitoring HCC recurrence [44]. Several studies, including our previous investigations, have demonstrated that CD44v6+ cells isolated from HCC cell lines exhibit increased self-renewal, proliferation, migration, and invasion as well as resistance to sorafenib and tumorigenic capacity [30]; however, the signal transduction cascades by which CD44v6 maintains HCC stemness responses have not previously been elucidated.

Met is involved in the tumorigenesis of various cancers and its phosphorylation activates downstream signaling cascades that can regulate cancer cell behavior. Binding of HGF to the SEMA domain of Met leads to receptor homodimerization, autophosphorylation of tyrosine residues in the tyrosine kinase domain, and activation of the downstream Ras/MAPK, PI3K/Akt, and Ras/Rac/Pho pathways [45]. Previous studies have elucidated that cJun has essential roles in hepatocyte survival and liver regeneration as well as promoting tumor initiation in carcinogenesis [46–48]. For example, in colorectal cancer, phospho-cJun is induced by Src, promotes invasion and metastasis in patients, and is associated with poor patient survival [49]. In breast cancer, activated cJun is predominantly expressed at the invasive front and is associated with proliferation and angiogenesis [50]. Further, Oh et al. found that regulation of the dynamic chromatin architecture of the epidermal differentiation complex is modulated by cJun/AP-1 binding and responds to developmental signals at the onset of differentiation in mouse embryos [51], while Ibrahim et al. showed that Nanog was overexpressed in colorectal cancers via cJun/TCF4-mediated transcriptional activation [34]. These results demonstrate that maintenance of cell stemness is regulated by cJun signaling; however, the exact mechanism underlying cJun activation in HCC CSCs was unclear. In our study, we extend the findings of previous investigations to identify the exact cJun binding site (1700 bp upstream of the Nanog TSS) using luciferase reporter and ChIP assays.

Nanog, a homeobox binding transcription factor, is essential for the maintenance of CSC pluripotency and self-renewal. In CSCs, Nanog induces metastasis, self-renewal, tumorigenesis, tumor relapse, and drug-resistance [21]. Jin et al. showed that USP21 is a specific Nanog deubiquitinating enzyme that leads to its degradation [52]. Further, Suzuki et al. showed that the mouse Nanog 5′ promoter region contains a STAT3 binding site 5 kb upstream of the TSS [53]. Moreover, Rodda et al. identified an Oct4/Sox2 motif −180 bp upstream of the TSS, which is important for Nanog regulation [54]. Here, we found that cJun could bind at −1700 bp upstream of the Nanog TSS, suggesting that Met regulates Nanog expression and function via regulation of cJun binding to the Nanog promoter region.

The orthotopic liver xenograft tumor model demonstrated that HGF/MET signaling plays a significant role in CD44v6+ HCC cells. Although the number of applications of this model in NOD/SCID mice is limited, our HCC cell line and clinical data further support our hypothesis. In the future, we aim to study additional animal models and clinical data from large studies to further verify and explore our conclusions.

The development of tyrosine-kinase inhibitors is an important facet of CSC research. Selective MET inhibitors, such as tepotinib [55, 56], capmatinib [57], and savolitinib [58], with promising survival benefits have emerged. Recently, tepotinib was approved for the treatment of unresectable and advanced non-small-cell lung cancer with METex14 mutation, making it the first MET-TKI to be approved worldwide [59]. We used the Met inhibitor, PHA665752, in CD44v6+ cells to demonstrate the importance of HGF/MET signaling in maintenance of CSC stemness. Overall, our findings reveal a new molecular mechanism involving Nanog signaling activated by Met in CD44v6+ CSCs. Our evidence suggests that cJun regulates Nanog expression and function by binding to a specific site in the Nanog promoter. Our findings reveal a potential new molecular therapeutic target in CD44v6+ CSCs.

## 5. Conclusions

**Figure 5:**
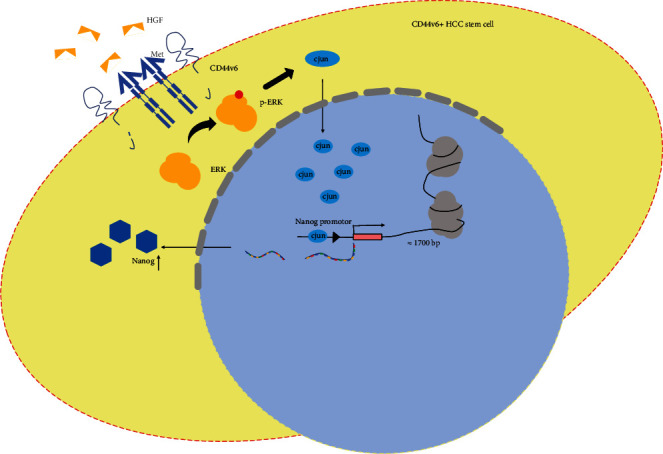
Schematic illustration of the mechanism by which the transcription factor cJun is binding in the transcription start site of Nanog and then promotes the Nanog expression, contributing to the maintenance of stemness of CD44v6+ HCC cells.

Our study demonstrates that the HGF/MET signaling pathway plays an important role in maintenance of CD44v6+ HCC cell stemness by enhancing cJun transcription factor activity. Mechanistically, cJun binds 1700 bp upstream of the Nanog TSS to promote Nanog expression, contributing to the maintenance of CD44v6+ HCC cell stemness (Figure 5).

## Data Availability

All data that support the findings in this study are available from the corresponding author upon reasonable request. Some data may not be made available because of privacy or ethical reasons.

## Supplementary Materials

Supplementary Materials Figure S1: (A) Flow cytometry analysis shows the expressions of CD44v6 in several cell lines, respectively. The expression of CD44v6 was almost 56% in SNU398 cell lines. (B) The immunohistochemical staining shows the expressions of CD44v6 and Nanog in surgical specimen from patients with HCC. The expression of CD44v6 and Nanog in tumor tissue was significantly higher than in adjacent nontumor tissue. Scale bar, 20 *μ*m. (C) and (D) qPCR showed that the expression of EMT-related genes and stemness-associated genes were extraordinarily decreased in mRNA level between CD44v6 positive and negative cells. ^∗∗∗^*p* < 0.001, ^∗∗∗∗^*p* < 0.0001, *t*-test. (E) Representative images of immunohistochemical staining of Met and Nanog in NC group and shMet group in subcutaneously xenografted tumors of mice. Scale bar, 50 *μ*m. (F)Western blot was performed in CD44v6+ SNU398 cells, which were treated with PHA665752 1uM for 24 hours. The expression of Met and AKT was downregulated in CD44v6+ HCC cells with PHA665752. *β*-Actin was used as a normalized control. (G) The stemness relative genes, including Nanog, Sox2, and Oct4, were also downregulated in CD44v6+ HCC cells with PHA665752. *β*-Actin was used as a normalized control. (H) The EMT relative genes, including E-cadherin, Vimentin, and Snail1, were also decreased in CD44v6+ HCC cells with PHA665752. *β*-Actin was used as a normalized control. Figure S2: (A) The MAPK signal pathway relative genes ERK and the p-ERK were decreased in CD44v6+ HCC cells with shMet. *β*-Actin was used as a normalized control. (B) and (C) qPCR showed that the expression of stemness associated genes and EMT-related genes were decreased in mRNA level in shMet group. ns. non sence, ^∗^*p* < 0.05, ^∗∗^*p* < 0.01, ^∗∗∗^*p* < 0.001, ^∗∗∗∗^*p* < 0.0001, *t*-test. Table S1 Caption: The sequences of primer in this research. Table S2 Caption: The ChIP PCR primer sequence (c-Jun binds to the promoter of Nanog).
