# Supplementary figures and images for: CD44v6+ Hepatocellular Carcinoma Cells Maintain Stemness Properties through Met/cJun/Nanog Signaling
Source: Stem Cells Int. 2022 Nov 7;2022:5853707. doi: 10.1155/2022/5853707 (PMC9663228; doi:10.1155/2022/5853707)

**A**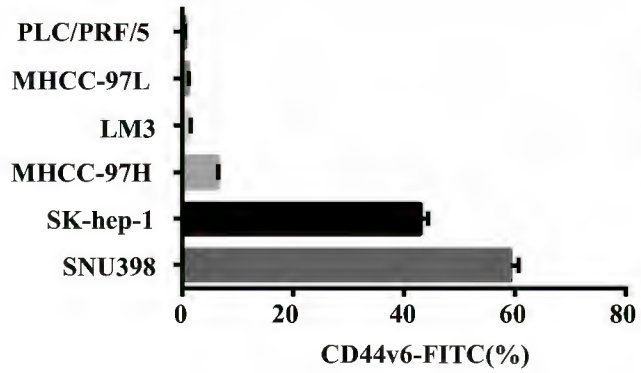**B**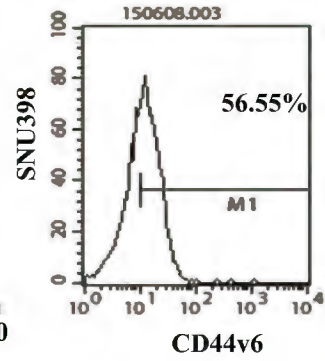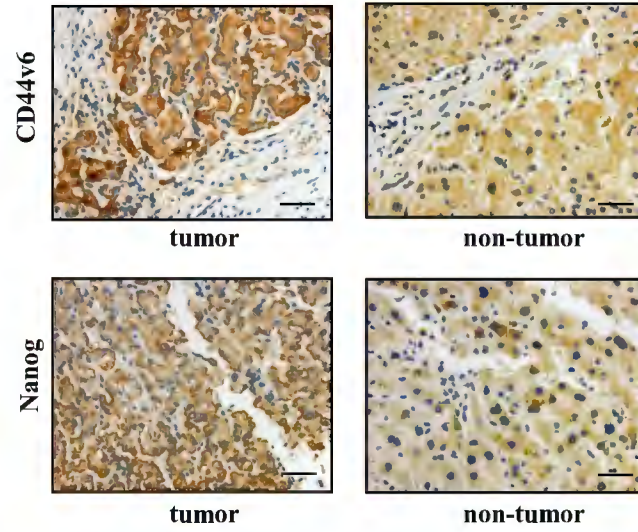**C**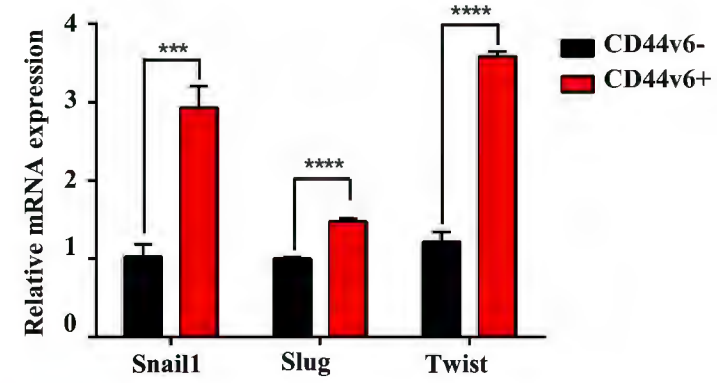**D**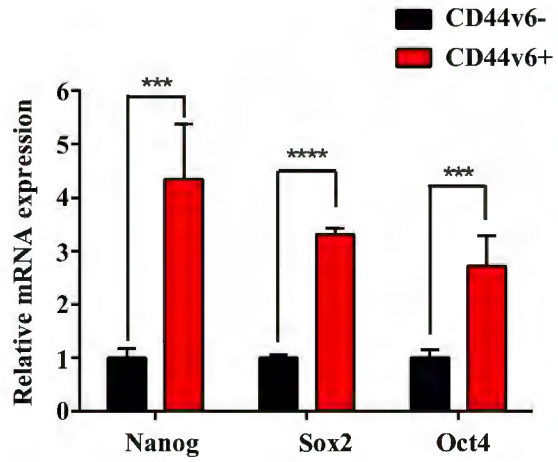**E**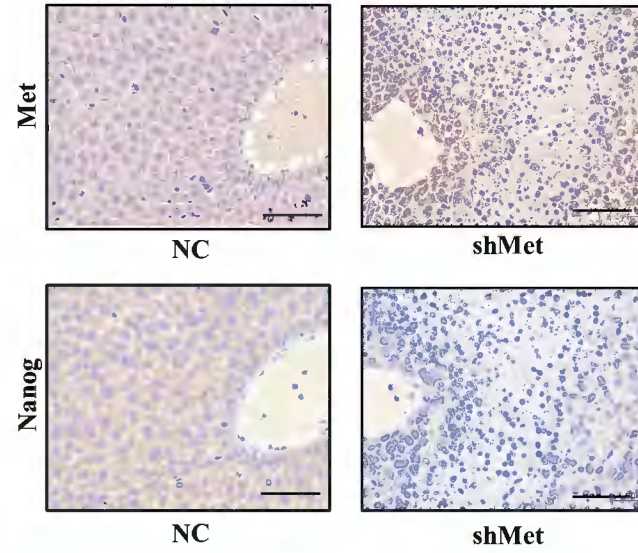**F**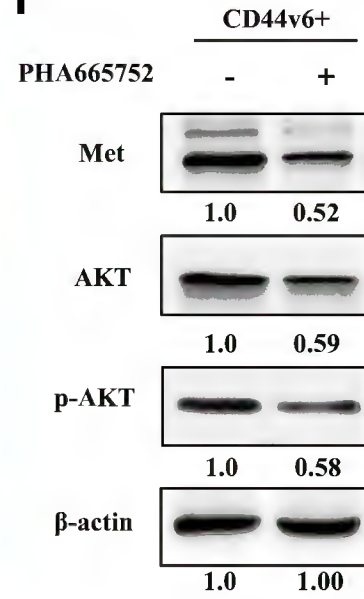**G**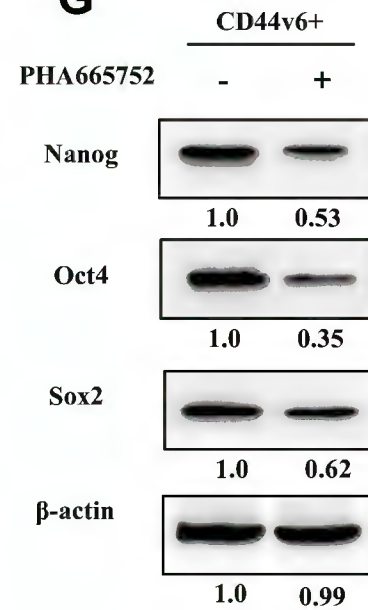**H**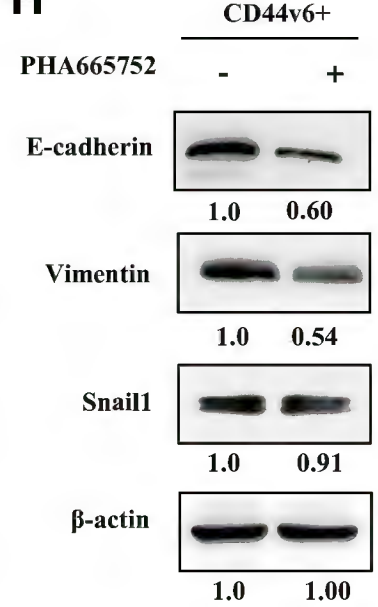

Supplement: Supplementary Materials — Figure S1: (A) Flow cytometry analysis shows the expressions of CD44v6 in several cell lines, respectively. The expression of CD44v6 was almost 56% in SNU398 cell lines. (B) The immunohistochemical staining shows the expressions of CD44v6 and Nanog in surgical specimen from patients with HCC. The expression of CD44v6 and Nanog in tumor tissue was significantly higher than in adjacent nontumor tissue. Scale bar, 20 μm. (C) and (D) qPCR showed that the expression of EMT-related genes and stemness-associated genes were extraordinarily decreased in mRNA level between CD44v6 positive and negative cells. ∗∗∗p < 0.001, ∗∗∗∗p < 0.0001, t-test. (E) Representative images of immunohistochemical staining of Met and Nanog in NC group and shMet group in subcutaneously xenografted tumors of mice. Scale bar, 50 μm. (F)Western blot was performed in CD44v6+ SNU398 cells, which were treated with PHA665752 1uM for 24 hours. The expression of Met and AKT was downregulated in CD44v6+ HCC cells with PHA665752. β-Actin was used as a normalized control. (G) The stemness relative genes, including Nanog, Sox2, and Oct4, were also downregulated in CD44v6+ HCC cells with PHA665752. β-Actin was used as a normalized control. (H) The EMT relative genes, including E-cadherin, Vimentin, and Snail1, were also decreased in CD44v6+ HCC cells with PHA665752. β-Actin was used as a normalized control. Figure S2: (A) The MAPK signal pathway relative genes ERK and the p-ERK were decreased in CD44v6+ HCC cells with shMet. β-Actin was used as a normalized control. (B) and (C) qPCR showed that the expression of stemness associated genes and EMT-related genes were decreased in mRNA level in shMet group. ns. non sence, ∗p < 0.05, ∗∗p < 0.01, ∗∗∗p < 0.001, ∗∗∗∗p < 0.0001, t-test. Table S1 Caption: The sequences of primer in this research. Table S2 Caption: The ChIP PCR primer sequence (c-Jun binds to the promoter of Nanog). [file 5853707.f1.zip › Sup Fig1 (1).pdf]

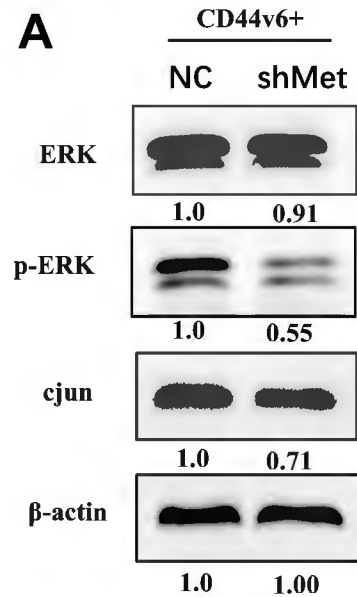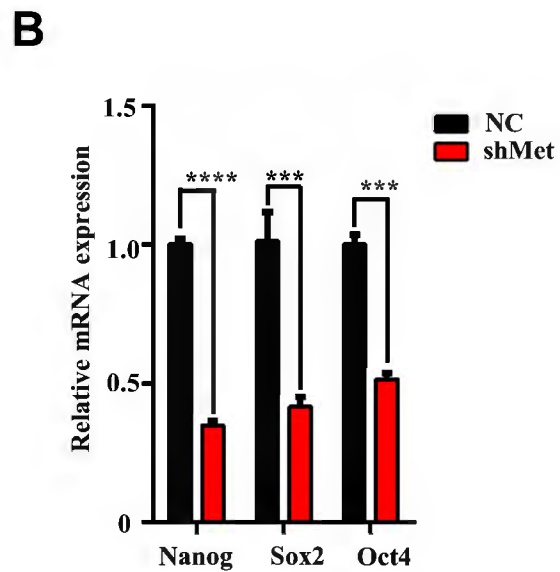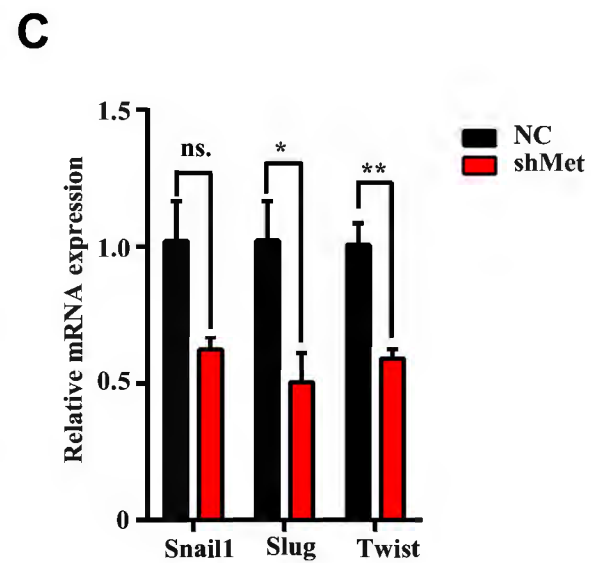

Supplement: Supplementary Materials — Figure S1: (A) Flow cytometry analysis shows the expressions of CD44v6 in several cell lines, respectively. The expression of CD44v6 was almost 56% in SNU398 cell lines. (B) The immunohistochemical staining shows the expressions of CD44v6 and Nanog in surgical specimen from patients with HCC. The expression of CD44v6 and Nanog in tumor tissue was significantly higher than in adjacent nontumor tissue. Scale bar, 20 μm. (C) and (D) qPCR showed that the expression of EMT-related genes and stemness-associated genes were extraordinarily decreased in mRNA level between CD44v6 positive and negative cells. ∗∗∗p < 0.001, ∗∗∗∗p < 0.0001, t-test. (E) Representative images of immunohistochemical staining of Met and Nanog in NC group and shMet group in subcutaneously xenografted tumors of mice. Scale bar, 50 μm. (F)Western blot was performed in CD44v6+ SNU398 cells, which were treated with PHA665752 1uM for 24 hours. The expression of Met and AKT was downregulated in CD44v6+ HCC cells with PHA665752. β-Actin was used as a normalized control. (G) The stemness relative genes, including Nanog, Sox2, and Oct4, were also downregulated in CD44v6+ HCC cells with PHA665752. β-Actin was used as a normalized control. (H) The EMT relative genes, including E-cadherin, Vimentin, and Snail1, were also decreased in CD44v6+ HCC cells with PHA665752. β-Actin was used as a normalized control. Figure S2: (A) The MAPK signal pathway relative genes ERK and the p-ERK were decreased in CD44v6+ HCC cells with shMet. β-Actin was used as a normalized control. (B) and (C) qPCR showed that the expression of stemness associated genes and EMT-related genes were decreased in mRNA level in shMet group. ns. non sence, ∗p < 0.05, ∗∗p < 0.01, ∗∗∗p < 0.001, ∗∗∗∗p < 0.0001, t-test. Table S1 Caption: The sequences of primer in this research. Table S2 Caption: The ChIP PCR primer sequence (c-Jun binds to the promoter of Nanog). [file 5853707.f1.zip › Sup Fig2.pdf]
